# Enhancing dexterity: Soft pneumatic actuation utilizing granular jamming for a human finger flexo-extension

**DOI:** 10.1017/wtc.2024.29

**Published:** 2025-03-03

**Authors:** X. Yamile Sandoval-Castro, J. German Cortes-Gonzalez, Maximiano F. Ruiz-Torres, Eduardo Castillo-Castaneda, Med Amine Laribi

**Affiliations:** 1 Department of Mechatronics, School of Engineering and Sciences, Tecnologico de Monterrey, Santiago de Querétaro, México; 2 Department of Mechatronics, Centro de Investigación en Ciencia Aplicada y Tecnología Avanzada Unidad Querétaro, Instituto Politécnico Nacional, Santiago de Querétaro, México; 3 Department of GMSC, Pprime Institute CNRS, UPR 3346, ISEA-ENSMA, University of Poitiers, Poitiers, France

**Keywords:** bending motion, bioinspired design, flexo-extension of a human finger, force characterization, granular jamming, pneumatic soft actuator, stiffness tuning

## Abstract

This article presents a bioinspired pneumatic soft actuator designed to mimic the flexo-extension movement of the human finger, with a particular focus on stiffness modulation through granular jamming. Three-chamber geometries – honeycomb, rectangular, and half-round – were evaluated to optimize curvature performance, utilizing Mold Star 15 Slow elastomer for actuator fabrication. Granular jamming, both passive and active, was implemented within the inextensible layer using chia and quinoa grains to enhance stiffness modulation. Experimental results revealed that the honeycomb geometry most closely aligned with the natural index finger trajectory. Stiffness evaluations demonstrated a range of 0–0.47 N/mm/° for quinoa and 0–0.9 N/mm/° for chia. The actuator’s force output increased by 16% for quinoa and 71% for chia compared to the nonjammed configuration. This enhanced performance is particularly beneficial for applications such as hand rehabilitation, where adaptive stiffness and force modulation are critical. Granular jamming, especially with active chia, provided superior adaptability for tasks requiring variable stiffness and resistance, making it a promising candidate for wearable robotic applications in rehabilitation.

## 1. Introduction

Soft robotics has emerged as a transformative approach in robotic design, emphasizing flexible, elastic materials that allow for safer and more adaptable interactions with humans and diverse environments. While traditional rigid robots face challenges in tasks that require high precision and force in controlled settings, they often face limitations in dynamic or unstructured environments due to their inflexibility. In contrast, soft robots, particularly those using pneumatic soft actuators, offer advantages such as high compliance and dexterity, bioinspired movement, environmental adaptability, and safe human interaction, making them well-suited for complex applications like medical rehabilitation and assistive robotics.

Despite these advantages, a key challenge remains: most soft actuators lack the ability to exert sufficient force or modulate stiffness effectively, limiting their application in tasks requiring precision or significant strength, such as object manipulation and gripping. This challenge is particularly evident in medical applications, where soft robotics innovations have proven promising but still fall short in certain areas. For example, robotic gloves designed to improve grasp capabilities and assist in rehabilitation have been developed by Alicea et al. (2021), Thalman et al. (2022), and Proietti et al. (2024), yet they lack the capacity for stiffness tuning necessary for more dynamic tasks. Cao et al. (2021) demonstrated the potential of combining pneumatic actuators with granular jamming to enhance force output in a robotic glove, while Polygerinos et al. (2015) introduced a soft glove that achieves flexo-extension of the fingers. These studies show the potential of soft robots in healthcare but highlight the need for improved stiffness-tuning mechanisms to handle complex, high-force tasks.

Among soft pneumatic actuators, bending motion has gained significant attention due to its simplicity and versatility, particularly in applications requiring safe and adaptive interactions with humans and objects. While studies have demonstrated bending actuators capable of achieving constant curvature (Santacruz-Mondragon et al., 2023; Khalil et al., 2024; Allen et al., 2024; Yu and Fu, 2023), achieving nonhomogeneous or variable curvature remains a key challenge. Such curvature adaptability is essential for tasks that demand shape conformation, like medical rehabilitation, where actuators must closely follow and support complex anatomical contours. Recent approaches, such as those by Song et al. (2021) and Hashemi et al. (2021), have explored bending actuators that better conform to object shapes, enhancing adaptability. For instance, Yang et al. (2020) proposed a bioinspired actuator combining solid sections with air chambers, allowing selective bending in specific areas, which improves control and flexibility. Similarly, Luo et al. (2022) advanced actuator design using machine knitting to create complex shapes, including L- and S-curves, for applications in assistive devices. Despite these advancements, current designs still fall short in providing both the sufficient force output and stiffness modulation necessary for demanding applications like rehabilitation, where precise, dynamic shape adaptability is crucial.

Stiffness modulation is crucial to advancing the functionality of soft actuators, especially for tasks like grasping and rehabilitation. To address this, various techniques have been investigated, including granular jamming, laminar jamming, and thermal methods. Granular jamming, a core focus of this study, allows actuators to transition between fluid-like and rigid states by manipulating granular materials inside the actuator (Hu et al., 2022). The choice of granular material impacts stiffness performance, as shown by experiments using ground coffee beans (Cardin-Catalan et al., 2022), rice (Abeach et al., 2018; Gao et al., 2021), and iron grains (Clerc et al., 2024), with smaller particles generally offering a greater range of stiffness due to tighter packing and frictional forces as demonstrated (Yang et al., 2020; Aktaş et al., 2021). This versatility, combined with the ability to offer on-demand stiffness adjustments, makes granular jamming particularly advantageous for dynamic applications requiring high adaptability. In contrast, laminar jamming involves layered structures that create stiffness through interlayer friction when pressure is reduced as was shown by Mukaide et al. (2020), Shah et al. (2021), Shen et al. (2020), and Mészáros and Sárosi (2023). While this method provides uniform stiffness, its limited range of modulation restricts its effectiveness in tasks needing significant stiffness variation. On the other hand, thermal methods alter stiffness by changing the elasticity of thermoplastics at elevated temperatures. Though they can achieve notable stiffness changes, they are less responsive than pressure-based methods and can be energy-intensive, which limits their applicability in real-time, dynamic environments (Yan et al., 2022; Luong et al., 2022; Ma et al., 2022. Given these considerations, granular jamming was selected for this study due to its simplicity, adaptability, and broader range of stiffness modulation, which are essential for developing versatile soft-robotic systems.

Regarding rehabilitation tasks for hand flexo-extension, the literature indicates that the required force range is between 1 and 2 N (Bouzit et al., 2002; Kawasaki et al., 2005). Most soft actuators using elastomeric materials achieve forces below 1 N, which may limit their applicability in such tasks. However, stiffness modulation through jamming has demonstrated a significant performance improvement, with actuators achieving at least 50% more force compared to configurations without jamming, as reported by Aktaş et al. (2021) and Mészáros and Sárosi (2023). This highlights the effectiveness of jamming mechanisms in enhancing force output for applications requiring dynamic adaptability.

This study introduces an innovative approach combining active and passive granular jamming to modulate stiffness in a soft pneumatic actuator inspired by the flexo-extension motion of a human index finger. We designed three distinct chamber geometries – honeycomb, half-round, and rectangular – and tested natural grains (chia and quinoa) as environmentally friendly jamming materials. These configurations enable the actuator to achieve superior stiffness during flexo-extension, increasing force output for tasks like medical rehabilitation.

The contribution of this work lies in the innovative integration of granular jamming with bioinspired soft actuator designs featuring distinct chamber geometries (honeycomb, rectangular, and half-round). Additionally, this study uniquely employs natural, environmentally friendly grains (chia and quinoa) as jamming materials and provides comprehensive experimental validation of stiffness modulation in both active and passive configurations. This approach addresses limitations of prior studies, such as insufficient force output and adaptability to complex trajectories, advancing the field of soft robotics with potential applications in rehabilitation, human–robot interaction, and adaptive industrial tasks.

## 2. Finger-bending soft actuator design and modeling

### 2.1. Bioinspired soft actuator design mimicking index finger flexion–extension

The design of the soft actuator reported in this article is based on the trajectory of a human index finger, specifically intended to facilitate bending for medical rehabilitation tasks.

**Figure 1.**
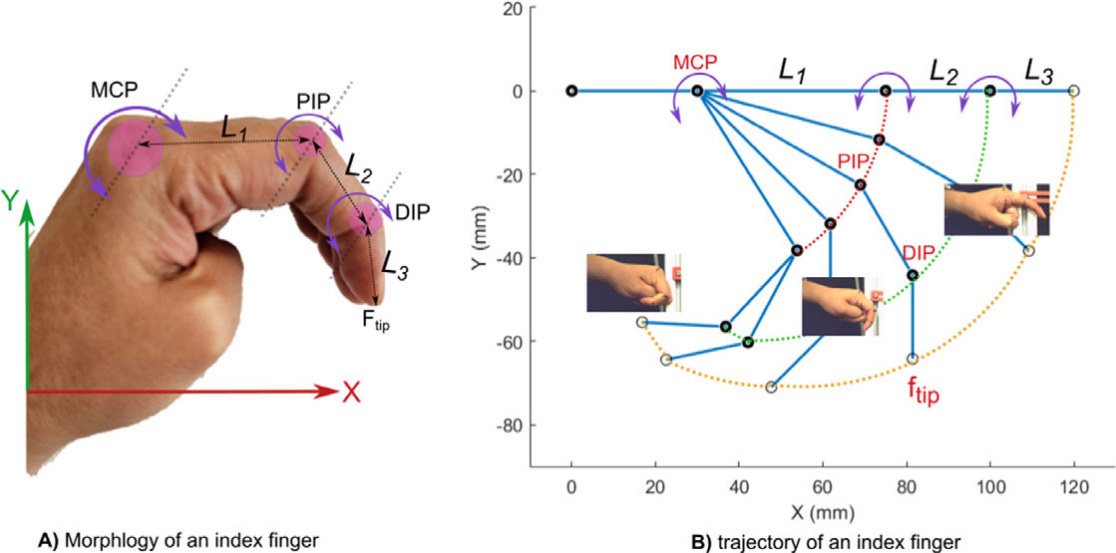
Characterization of the trajectory and dimensions of a human index finger: A) Trajectory ofone of the author’s index finger. B) Morphology of a human index finger.

Figure 1a illustrates the morphology of an index finger, which has three rotational joints that allow flexo-extension motion on the *XY* plane. These joints are identified as the metacarpophalangeal (MCP), proximal interphalangeal (PIP), and distal interphalangeal (DIP) joints, according to Xu et al. (2012).

To design the actuator, the flexion–extension trajectory of a volunteer’s index fingers was characterized using computer vision, and the dimensions of the phalanges were estimated ( 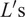 ). Figure 1b shows a graphical representation of the flexion–extension trajectory in various positions. The estimated geometric parameters are 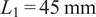 , 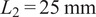 , and 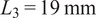 .

Based on the analysis of the flexion–extension trajectory, we proposed a segmented chamber design to achieve bending similar to that of an index finger. Chamber geometry, however, plays a crucial role in determining the bending trajectory and curvature of soft actuators (Xu et al., 2012). This study evaluates and compares three-chamber geometries – (a) rectangular, (b) honeycomb, and (c) half-round – to assess each actuator’s tip trajectory, curvature, and resemblance to the natural motion of the human index finger. The soft actuator design incorporates these three geometries to allow a comprehensive comparison.

**Figure 2.**
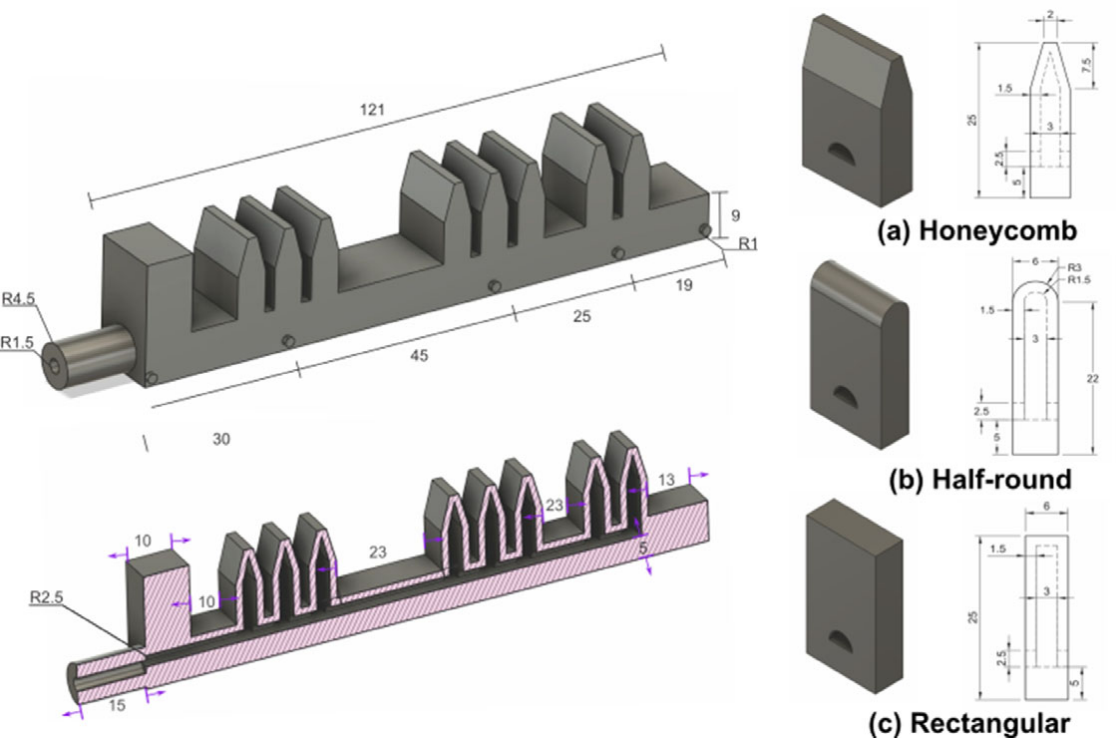
Diagrams (in mm) showing the dimensions of the proposed actuator and the honeycomb-shaped chambers.

As illustrated in Figure 2, the soft actuator measures 121 mm in length, 15 mm in width, and 25 mm in height, with a wall thickness of 1.5 mm. Additionally, 1 mm diameter markers were placed along each joint position for experimental tracking. Detailed chamber designs are provided for each geometry configuration.

### 2.2. Kinematic modeling

**Figure 3.**
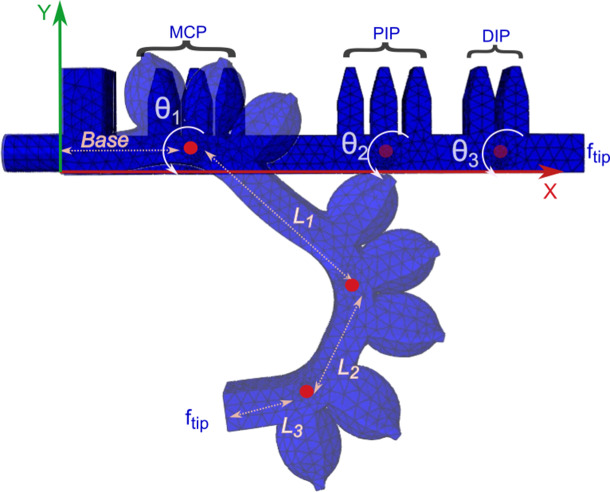
Design approach of the bending soft actuator.

In this configuration, applying air pressure causes only the targeted sections to bend, while the remaining structure retains its original shape. Three air chambers were placed over the MCP and PIP joints, and two chambers were positioned on the DIP joint due to its limited bending range compared to the other joints as shown in Figure 3. The three phalanges are labeled as *L*_1_, *L*_2_, and *L*_3_.

This design treats each phalangeal joint as rotational, with the segments considered as rigid lengths. With these considerations, the kinematic model can be developed using the Denavit–Hartenberg convention. The model for the fingertip ( 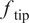 ) is described by the following equations, both when there is no pneumatic actuation ( 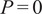 ) and when the pressure is greater than 0 ( 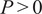 ).

When 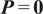



(1)

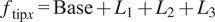



(2)

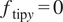



When 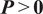



(3)

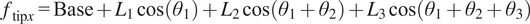



(4)

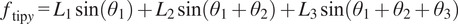



### 2.3. Manufacturing

The soft actuator was manufactured using a casting method, utilizing the elastomer Mold Star 15 Slow, which features an elongation of up to 440%. The actuator was built with three distinct geometries: rectangular, honeycomb, and half-round. Additionally, this study proposes stiffness modulation through granular jamming, which is detailed in the following section.

**Figure 4.**
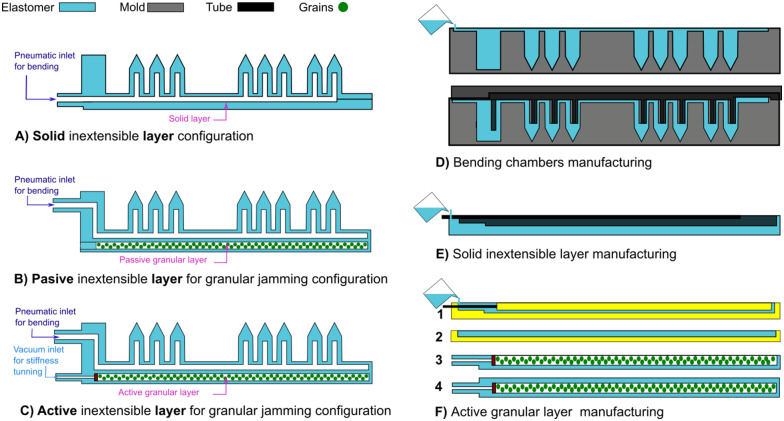
Casting process for soft actuators, illustrating the configurations for solid, passive, and active inextensible layers.

Figure 4a presents a diagram of the soft actuator with a classic solid inextensible layer. Figure 4b illustrates the soft actuator with a passive layer for granular jamming, where granular material is enclosed within a passive chamber. Finally, Figure 4c depicts the soft actuator with an active layer for granular jamming; this configuration includes two pressure inlets: the first one (positive pressure) for controlling the bending motion and the other for tuning stiffness via vacuum.

The following steps are required to manufacture the *pneumatic chambers*, which are consistent across all chamber geometries and inextensible configurations.

- **Pour elastomer into Mold 1**: fill Mold 1 completely with elastomer, as shown in the upper part of Figure 4d.
- **Align Mold 2 with Mold 1**: carefully position Mold 2 over Mold 1, ensuring that it displaces the elastomer to create hollow sections that will serve as air channels, as illustrated in the lower part of Figure 4d. Secure the two molds together using screws and nuts, and let the assembly dry according to the manufacturer’s recommended minimum drying time (4 hr).

To manufacture the *solid inextensible layer configuration*, position the tube inside the mold and pour the elastomer as shown in Figure 4e. Allow the elastomer to cure for 4 hr. Carefully demold both the pneumatic chambers and the inextensible layer, and then apply elastomer to the bonding surfaces to assemble and finalize the prototype, as illustrated in Figure 4a. This process is consistent for the honeycomb, rectangular, and half-round geometries.

To manufacture the *passive inextensible layer for granular jamming configuration* and the *active inextensible layer for granular jamming configuration*, a base piece is created as shown in Figure 4f1 along with solid parts as illustrated in Figure 4f2. After curing these molds, the base is filled with granular material (Figure 4f3). The base and solid parts are then assembled and bonded using elastomer, following the same process as in previous steps. The primary difference between these configurations is that the *passive inextensible layer* does not include a vacuum inlet as shown in Figure 4b, whereas the *active inextensible layer* does as illustrated in Figure 4c.

The manufacturing of the *passive inextensible layer for granular jamming configuration* and the *active inextensible layer for granular jamming configuration* is necessary to create a piece base as shown in Figure 4f1 and a solid part as illustrated in Figure 4f2. After curing these molds, the base is filled with a granular material (Figure 4f3), and then both the base and the solid pieces are assembled and glued with an elastomer as in the previous steps (Figure 4). The difference between these configurations is that the passive inextensible layer does not have a vacuum inlet while the passive inextensible layer has one.

## 3. Stiffness modulation through granular jamming

Stiffness modulation is a fundamental feature in soft actuators, enabling them to adapt to varying tasks by transitioning between a compliant state for safe interactions and a rigid state for exerting force or maintaining specific positions. In this study, stiffness modulation is achieved through granular jamming, which utilizes natural grains as environmentally friendly materials to enhance performance.

On the other hand, research findings related to medical rehabilitation (Bouzit et al., 2002; Kawasaki et al., 2005) indicate that the force required to flex and extend hand fingers is ~1 –2 N.

**Figure 5.**
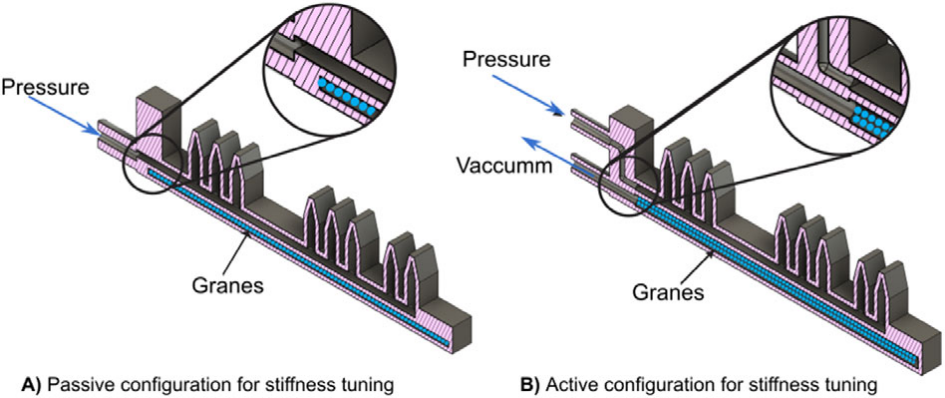
Cross-section view of the modified soft actuators for stiffness tuning.

To increment the exerted force by the actuators presented above, it was proposed to implement granular jamming in both ways, passive and active. Figure 5 shows an extra channel with dimensions 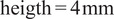 , 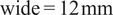 , and 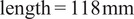 . For active jamming, a second air input was added to allow vacuum modulation.

### 3.1. Modeling of stiffness variation with granular jamming

The modeling of stiffness variation with granular jamming presented in this article is based on the analysis proposed by Yang et al. (2020).

**Figure 6.**
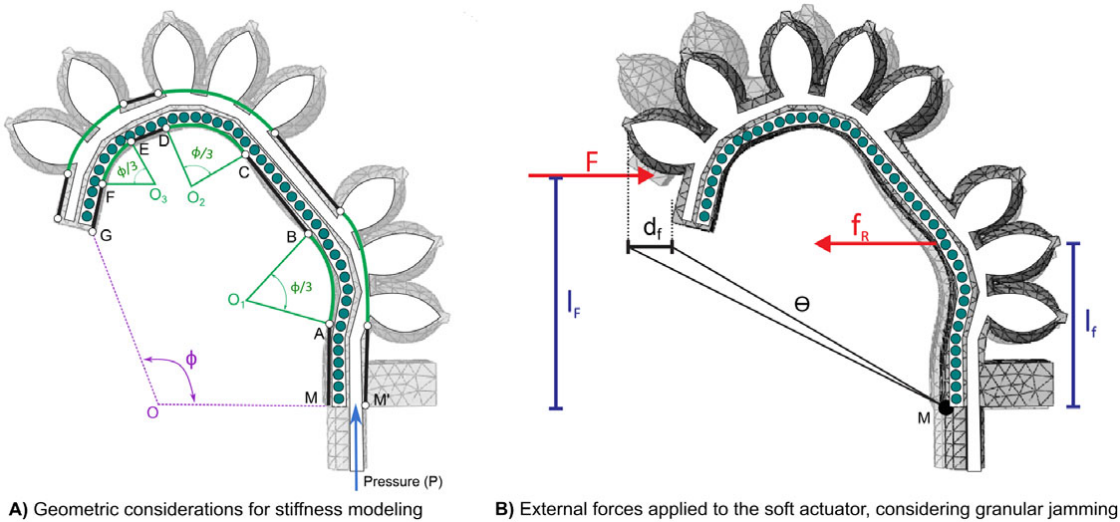
Diagram of stiffness modelling.

Figure 6 describes the soft actuator morphology, considering a bending curvature. Since the air chambers are not homogeneously distributed over the actuator, when air pressure is applied, some segments bend while others remain straight. To represent this, the lower and upper sides of the chamber have been divided into segments, using 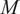 and 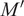 as the starting points and then going from 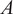 to 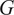 as needed. The curvature radius can be defined from the base to the tip 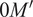 with an angle 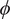 , while the arcs corresponding to each joint have an angle 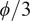 , as is described in Figure 6a.

The curvature radius 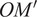 can be calculated using Eq. (5):
(5)

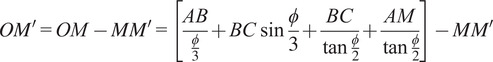



In this case, stiffness 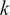 can be modeled as shown in Eq. (6).
(6)

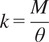

where 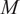 is the momentum applied to the actuator’s tip and 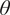 is the angle of the displacement caused by the 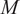 momentum. For this soft actuator, due to the existence of granular material inside the lower chamber, two momentums are applied, as shown in Figure 6b:

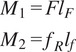



The displacement angle 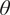 can be calculated based on the displacement of the tip 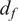 (Eq. 7), distance of the applied force 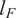 , bending angle 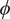 _,_ and curvature radius 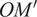 .
(7)

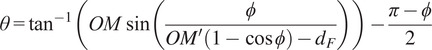



By analyzing the forces involved in the granular material and assuming spherical particles of the same size, the interaction force between particles, 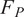 , can be expressed as a function of the applied pressure, 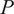 , due to the compression of the chamber caused by the bending motion. Additionally, it depends on the surface area, 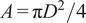 , where 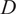 is the particle diameter. This relationship is described by Eq. (8).
(8)

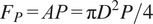



Considering the forces acting on a single particle, and assuming that each particle is surrounded by six others of the same size, the friction force can be calculated. With a friction coefficient 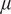 , the friction force is 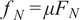 , where the normal force 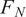 can be determined as 
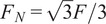
 . Thus, the friction force can be expressed as shown in Eq. (9).
(9)

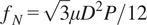



**Figure 7.**
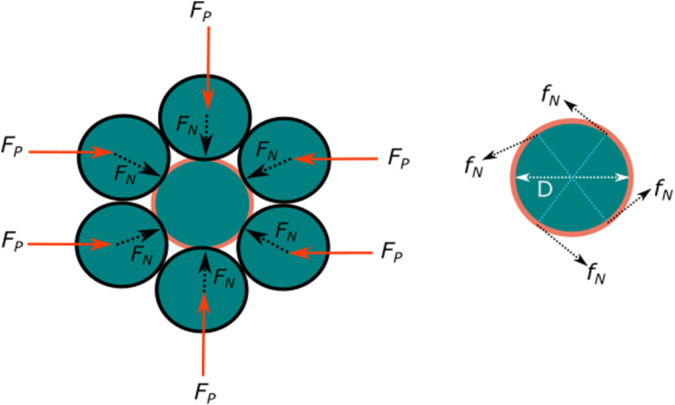
Forces on a spherical particle surrounded by identical particles.

As shown in Figure 7, four forces are acting over one particle. Considering the displacement of a particle 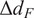 , the resulting work by these four forces is 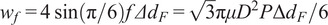 . Assuming the particles are stacked in layers and considering the total height of those layers as 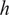 , 
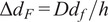
 can be calculated. Therefore, 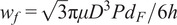 .

For each particle layer, the number of particles can be calculated using 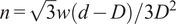 , where 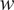 and 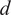 are the width and height of the cross-section of the chamber containing the particles, respectively. Assuming the number of layers 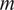 is given by 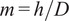 , the total work exerted by the particles is 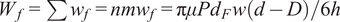 . The position of the resulting friction force 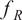 is assumed to be at half the height of the bent actuator, meaning 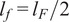 . The work can then be expressed as 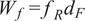 , leading to the resulting friction force as shown in Eq. (10)
(10)

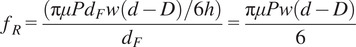



The torque generated by air pressure in the chambers can be calculated as 
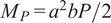
 , where 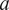 and 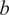 are the width and height of the cross-section of the air chamber, respectively. Assuming the torques in 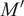 are balanced, 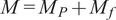 . Considering this, stiffness can then be calculated as shown in Eq. (11).
(11)

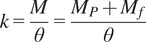



## 4. Characterization of soft actuator trajectories

### 4.1. Characterization of the soft actuator across three-chamber geometries

**Figure 8.**
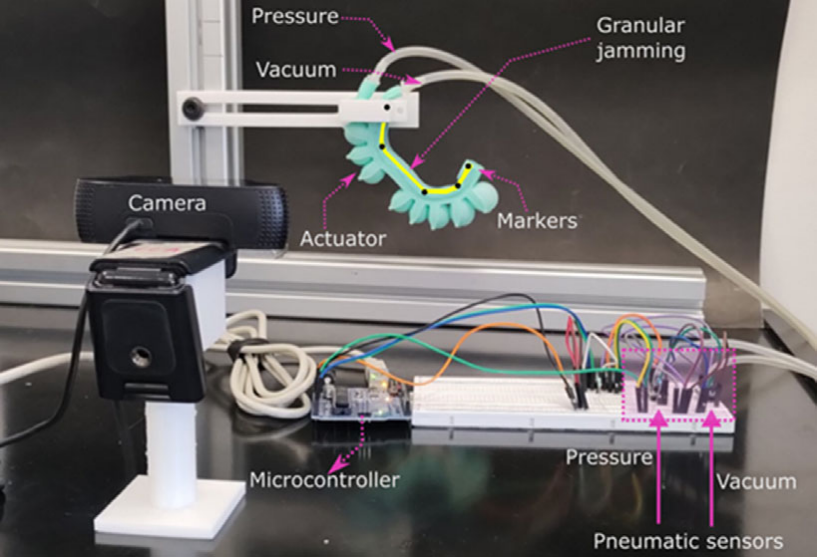
Experimental set-up for trajectories characterization.

The performance of the actuators with rectangular, half-round, and honeycomb chamber geometries was characterized using computer vision for the *solid inextensible layer configuration*, focusing on both trajectory and curvature. Figure 8 illustrates the experimental setup, where the actuator was fixed. Five black markers were placed on the inextensible layers to facilitate image tracking and estimate the position of the actuator tip and the lengths of the rigid sections. A webcam was employed to capture the data for characterization.

The objective of this characterization is to determine which chamber geometry most closely replicates the natural curvature and trajectory of the index finger, as shown in Figure 1a. For all experiments, seven tests were performed and the average results were reported. The maximum applied pressure for these tests was 45 kPa. The chamber geometry with the best performance will be chosen for replicating the flexo-extension of a human finger, focusing on applications in medical rehabilitation.

**Figure 9.**
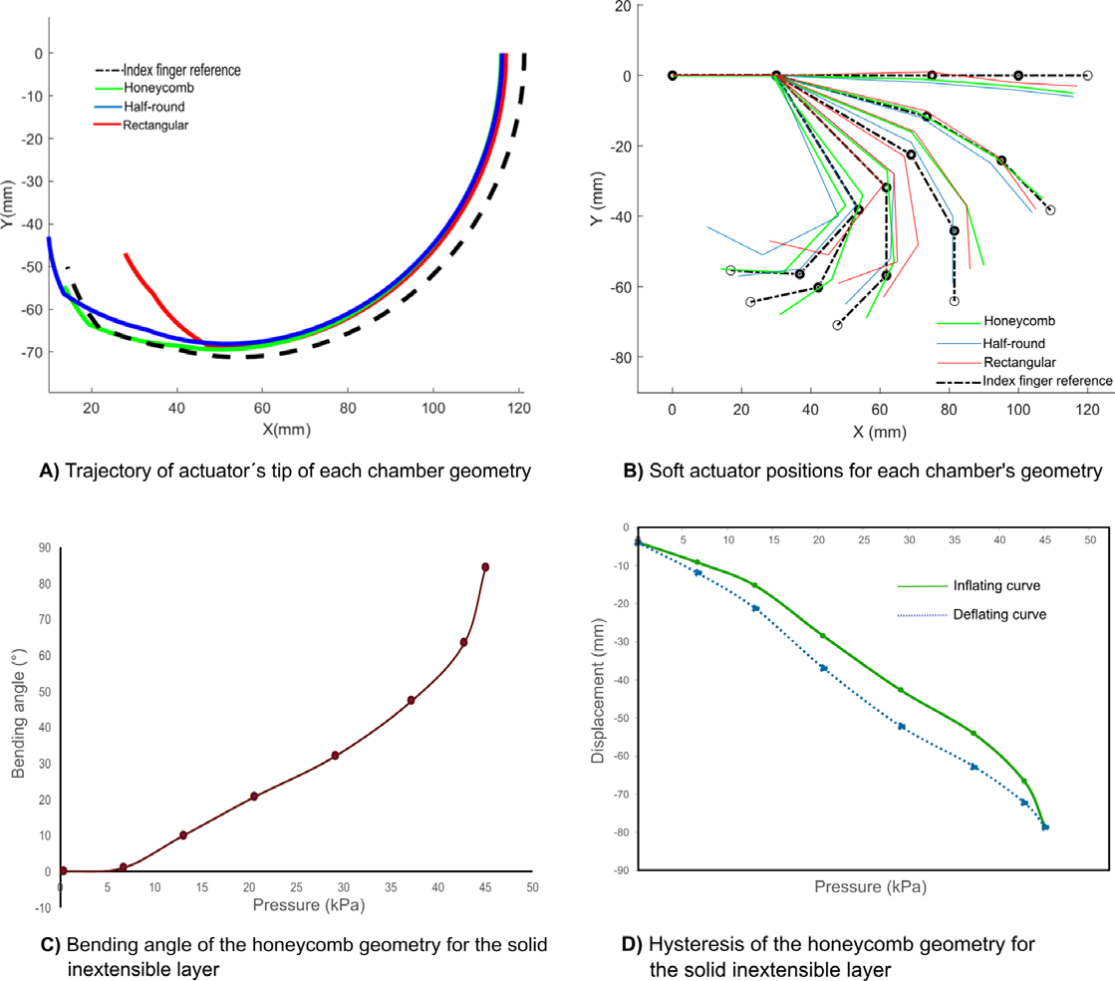
Performance of the actuator for each chamber geometry with the solid inextensible layer configuration.

Figure 9a shows the trajectories of each soft actuator geometry, estimated using computer vision and compared with the index finger trajectory (design reference depicted as a black dotted line) from Figure 1a. The plotted trajectories represent the average of seven tests for the soft pneumatic actuator across the three-chamber configurations. The computed standard deviations for the rectangular, half-round, and honeycomb geometries were 0.4, 0.49, and 0.33 (mm), respectively.

**Figure 10.**
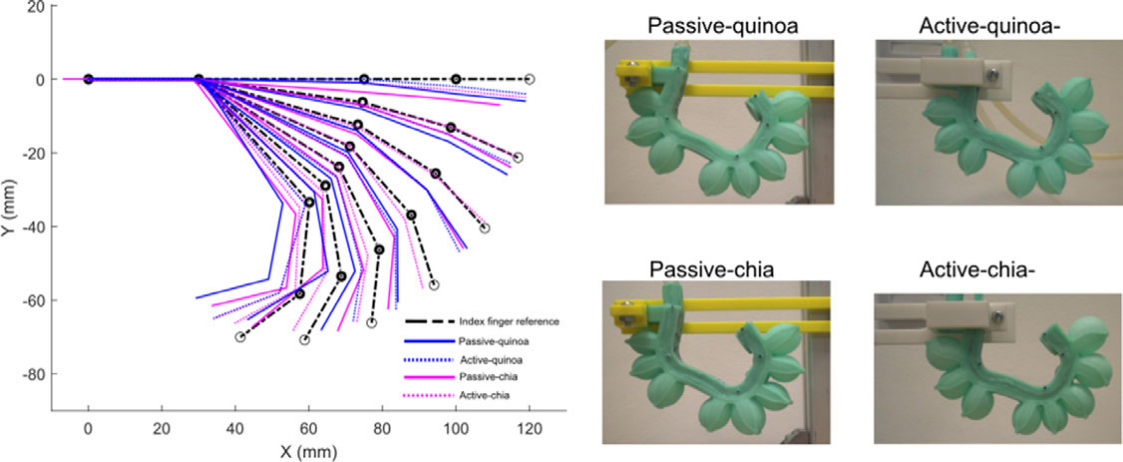
Trajectory of the tip of each actuator, considering granular jamming implementation.

Additionally, the phalangeal angles were estimated by tracking the markers shown in Fig. 10. To further analyze the behavior of the soft actuators, the phalangeal lengths were measured to assess their deformation along the trajectory, as the kinematic model assumes rigid links. The average lengths obtained were Base = 30.4 mm, 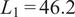 mm, 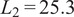 mm, and 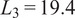 mm, with corresponding deformation percentages of 1.29%, 1.32%, 1.59%, and 2.43%, respectively. This experimental data was then used to compute the position of each phalangeal joint and the actuator tip, applying Eqs. (3) and (4) for the different chamber geometries.

The data were also utilized to visualize and compare the trajectories of the honeycomb, half-round, and rectangular soft pneumatic actuators against the design reference using MATLAB for virtual animation. As illustrated in Figure 9b, the honeycomb configuration not only closely follows the reference trajectory but also demonstrates superior performance in replicating the joint and link movements of the index finger compared to the other configurations.

The results shown in Figures 9a, b reveal that the honeycomb geometry (green line) aligns most closely with the reference, particularly at the final points of the trajectory. In contrast, the rectangular chamber geometry deviates significantly from the reference, exhibiting a tighter trajectory curvature, which suggests a more closed and restricted movement. On the other hand, the half-round geometry exhibits a trajectory with a longer curvature than the reference, making it less suitable for mimicking natural finger movements.

Based on these findings, the honeycomb chamber geometry is selected as the optimal design for flexo-extension tasks, especially in applications requiring accurate replication of human finger movements, such as medical rehabilitation.

Additionally, the experimental relationship between bending angle and pneumatic pressure was evaluated for the honeycomb chamber geometry, which most closely replicates the fingertip trajectory. This assessment was performed across pressures ranging from 0 to 45 kPa, with results averaged over seven tests. Figure 9c shows that the actuator reaches a maximum bending angle of 84.38°.

Hysteresis characteristics were also evaluated. Due to the silicone composition and stretchability of these soft actuators, their behavior forms a hysteresis loop, with distinct inflation and deflation paths as the pressure changes. The motion tracking system shown in Figure 8 monitored the actuator’s tip position in the *Y*-direction across seven cycles, with the average results reported. As illustrated in Figure 9d, the tip follows the solid-line curve during inflation and the dashed-line curve during deflation.

The curves in Figure 9d were generated by polynomial regression from discrete data points captured by the motion tracking system. Notably, the deflation curve has a steeper slope than the inflation curve, indicating that the actuator returns to the desired position more rapidly during deflation. This hysteresis cycle exhibits a typical pattern, as shown in Figure 9d.

### 4.2. Characterization of the soft actuator: passive and active layers

To investigate stiffness modulation, two organic granular materials, quinoa and chia, were tested in both passive and active inextensible layer configurations, utilizing the honeycomb chamber geometry. Two soft actuators with active inextensible layers were fabricated, referred to as *active chia* and *active-quinoa.* Similarly, two additional actuators with passive inextensible layers were manufactured, designated as *passive chia* and *passive quinoa.* This comparative approach highlights the significance of stiffness modulation, as the different materials and configurations allow for tailored adaptability and force output in soft actuators, critical for applications such as medical rehabilitation and human–robot interaction.

The curvature of the soft actuators with granular jamming was analyzed using the computer vision system illustrated in Figure 8. This system was employed to evaluate the variations in curvature resulting from both passive and active inextensible layer configurations for granular jamming.

In this analysis, seven tests were performed for each soft actuator. The average angles between each section and the link lengths were measured across all tests. Using this experimental data, the actuator’s tip position was calculated at seven different pressure levels (from 0 to 65 kPa) employing the forward kinematics model presented in Eqs. (3) and (4). The average link lengths obtained for the four configurations are presented in Table 1.

**Table 1.** Link lengths measured using computer vision and their deformation percentages for the soft actuators with granular jamming

Passive-chia		Active chia		Passive-quinoa		Active-quinoa	
Links	% Def.	Links	% Def.	Links	% Def.	Links	% Def.
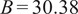	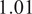	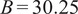	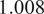	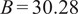	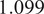	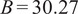	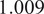
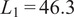	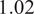	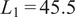	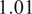	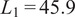	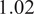	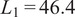	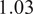
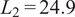	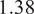	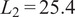	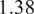	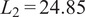	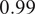	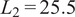	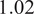
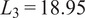	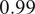	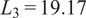	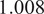	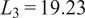	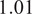	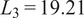	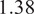

These data were used to visualize and compare the trajectories achieved by all the tested soft actuators. As shown in Figure 10, the soft actuators with granular jamming using chia, in both passive and active inextensible layer configurations, closely align with the trajectory of the human index finger. In contrast, actuators utilizing quinoa exhibit greater deviations, particularly in fully flexed positions. Moreover, the quinoa-based configurations display a higher percentage of deformation in their links, further highlighting the superior performance of the chia material for these applications.

Both configurations with the chia material were thoroughly characterized to determine the relationship between bending angle and pressure, as well as to analyze their hysteresis behavior.

**Figure 11.**
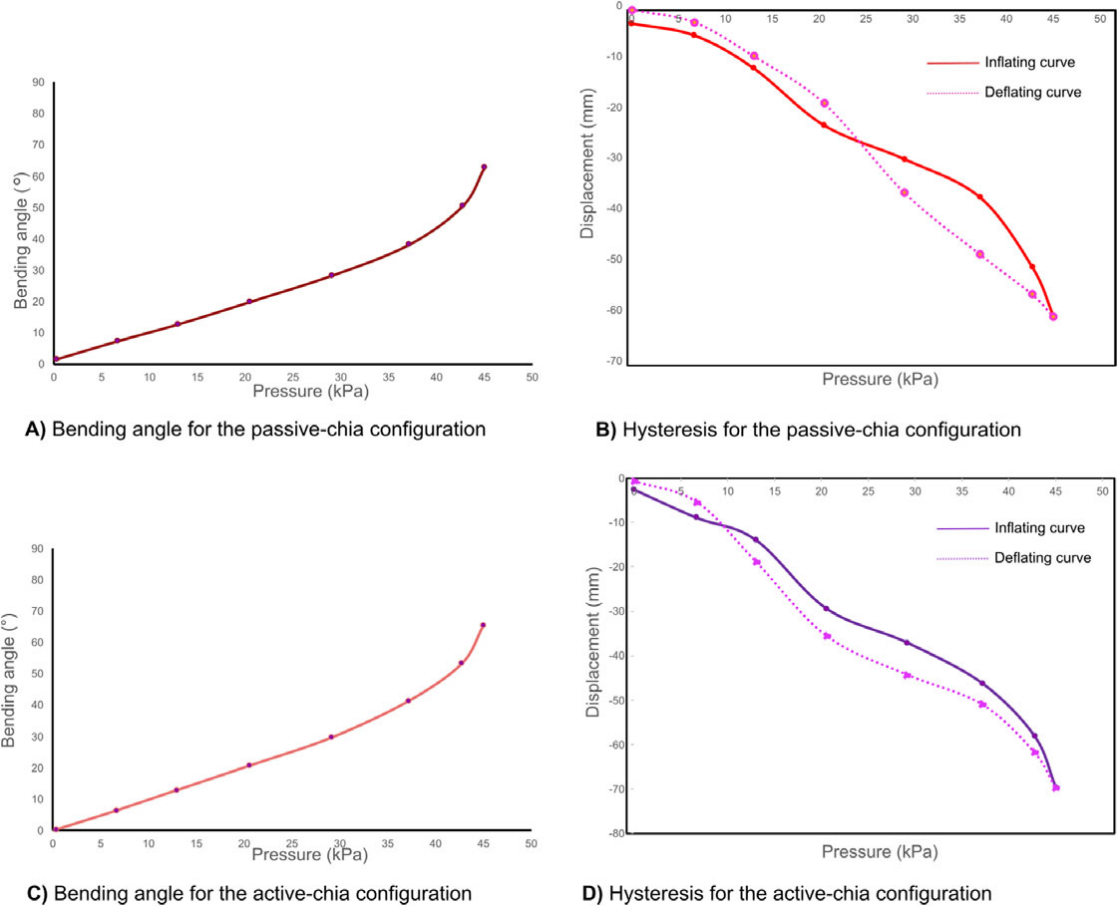
Performance of the soft actuator with the active–chia configuration, showcasing its bending angle response and hysteresis behavior.

The passive and active chia configurations achieved a bending angle of 62° at 65, as shown in Figures 11a, c. These configurations require higher pressure to achieve bending compared to the solid inextensible layer, indicating differences in their stiffness and response characteristics.

Regarding the hysteresis characteristics, in the granular jamming configuration, the deflation performance curves indicate that the actuator exhibits varying behaviors at pressure values closer to 0 kPa, as shown in Figures 11b, d, highlighting variability in its response, especially in the passive chia configuration.

## 5. Experimental validation of stiffness modulation

Granular jamming-based stiffness modulation is a critical aspect of the proposed actuator, allowing for improved force output and trajectory control. This section presents the experimental validation of stiffness modulation, focusing on its impact on actuator performance. The results highlight the benefits of both passive and active granular jamming configurations in achieving dynamic stiffness adjustment, which is essential for applications like rehabilitation and object manipulation.

### 5.1. Force characterization

An FSR 406 sensor was used to measure the force applied by the actuator at its tip. A set of weights ( 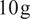 , 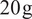 , 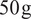 , 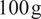 , 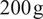 , and 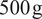 ) was used to calibrate the sensor.

**Figure 12.**
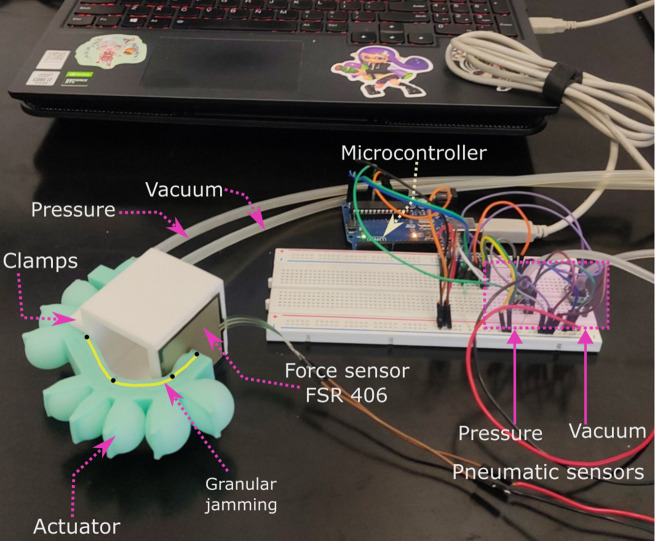
Experimental set-up for force characterization.

Force characterization was conducted using a controlled experiment, as illustrated in Figure 12. To emulate the action of pressing an object with a finger, the actuator grasped a cubic structure measuring 6 × 6 × 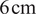 , with the FSR 406 sensor attached to one face. This setup ensured that during the bending movement, the actuator would press the sensor while holding the cube.

The force was characterized for the solid inextensible layer, as well as for both passive and active granular jamming configurations. For the solid and passive actuators, a positive pressure was applied and the resulting force was measured. For the active actuators, various vacuum levels ( 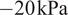 , 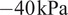 , and 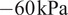 ) were applied across the entire positive pressure range ( 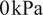 to 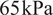 ). Seven tests were conducted for each configuration and the average results were reported.

**Figure 13.**
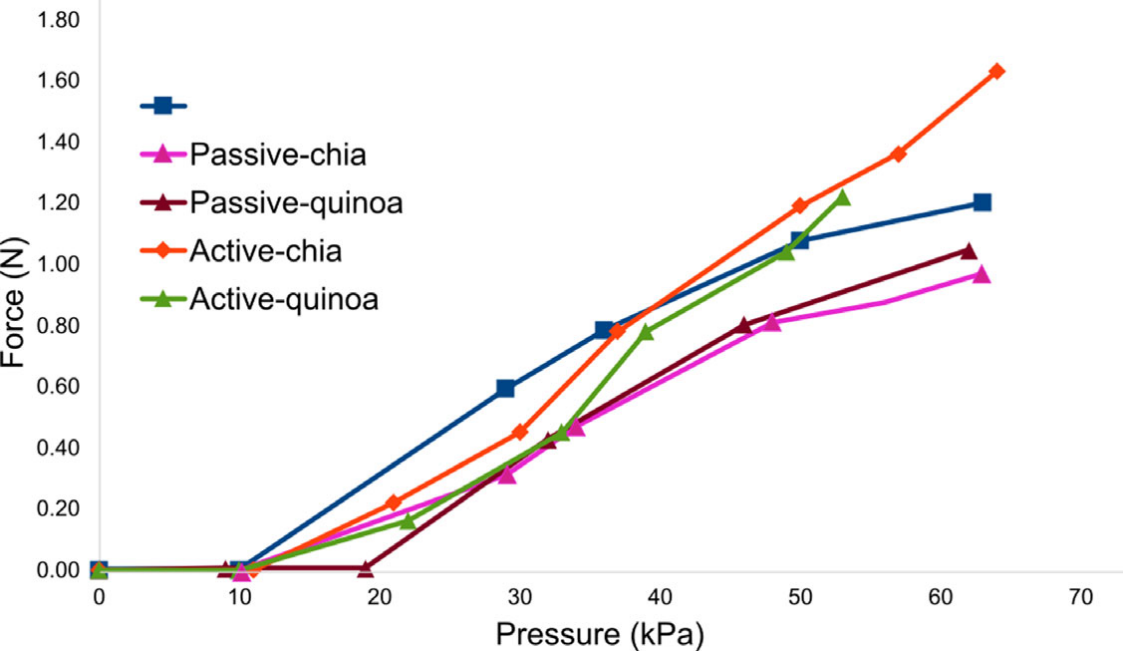
Graphics of force characterization for active and passive granular jamming.

The solid inextensible layer actuator achieved a maximum force of 1.2 N with a standard deviation of 0.2 (N). The passive actuators, passive chia and passive quinoa reduced the force to 0.97 and 1.05 N, respectively, with standard deviations of 0.58 (N) and 0.67 (N). In contrast, the actuators with active granular jamming showed an increase in force across different vacuum levels. As shown in Figure 13, at a vacuum level of 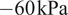 , the active quinoa actuator produced a force of 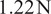 , while the active chia actuator reached 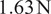 , with standard deviations of 0.21 and 0.43, respectively.

### 5.2. Experimental stiffness estimation

The rotational stiffness of the active granular jamming actuators ( 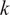 ) was estimated using experimental data and Eq. (11). The force ( 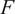 ) was computed experimentally as described in the previous section. The angle ( 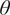 ) was calculated when the actuator bent to a certain position using computer vision, following the same methodology used to estimate the actuator’s trajectories. The distance ( 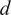 ) is controlled and is a known parameter.

Stiffness was estimated for the actuators with a positive pressure value of 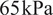 applied, and the vacuum levels were varied at 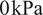 , 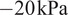 , 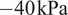 , and 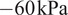 .

**Figure 14.**
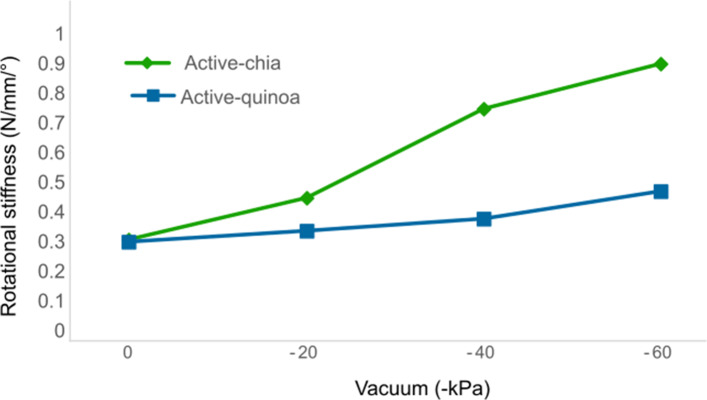
Estimated rotational stiffness for granular jamming, quinoa and chia seems.

Figure 14 presents the average results from seven tests for both actuators, demonstrating that the active chia actuator exhibits higher stiffness compared to the active quinoa actuator, with standard deviations of 0.21 and 0.39 (N/mm/°), respectively.

### 5.3. Flexo-extension of a human index finger

We implemented the soft actuator in a flexo-extension task for a human index finger to demonstrate its potential as a wearable device. The actuator was tested in three configurations: solid inextensible layer, and active granular jamming using chia and quinoa as granular materials. These experiments allowed us to evaluate the variations in exerted force across the configurations, with a particular focus on the enhanced force output achieved through active granular jamming. The results highlight the actuator’s effectiveness in mimicking natural finger movement and its suitability for wearable applications, especially in rehabilitation tasks.

For this test, the FSR402 sensor was secured to the finger and the soft actuator was placed over the sensor. The participant, who is the same individual involved in the link dimension design, was instructed to rest their hand on a wooden cane and remain passive throughout the test, to ensure consistent and accurate measurements.

**Figure 15.**
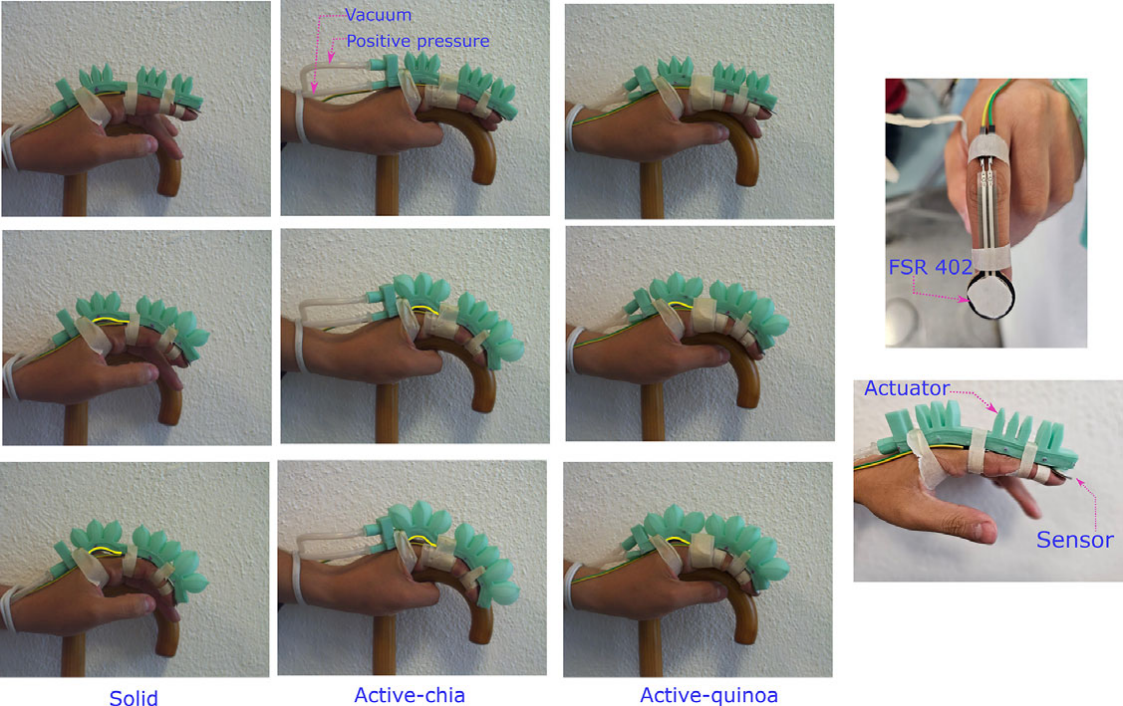
Flexo–extension of a human index finger, using soft actuators with granular jamming.

In this experimental evaluation, 10 tests were conducted and the average of the force measurements was reported. Figure 15 illustrates the process for three different levels of positive pressure in the solid inextensible layer, active chia, and active quinoa for granular jamming configurations. In Figure 15, it can be observed that as the pressure increases, the granular material undergoes reaccommodation in response to the vacuum. This leads to a curvature (highlighted in yellow) in the first link of the soft actuator, which is a result of how the actuator was attached to the hand.

**Figure 16.**
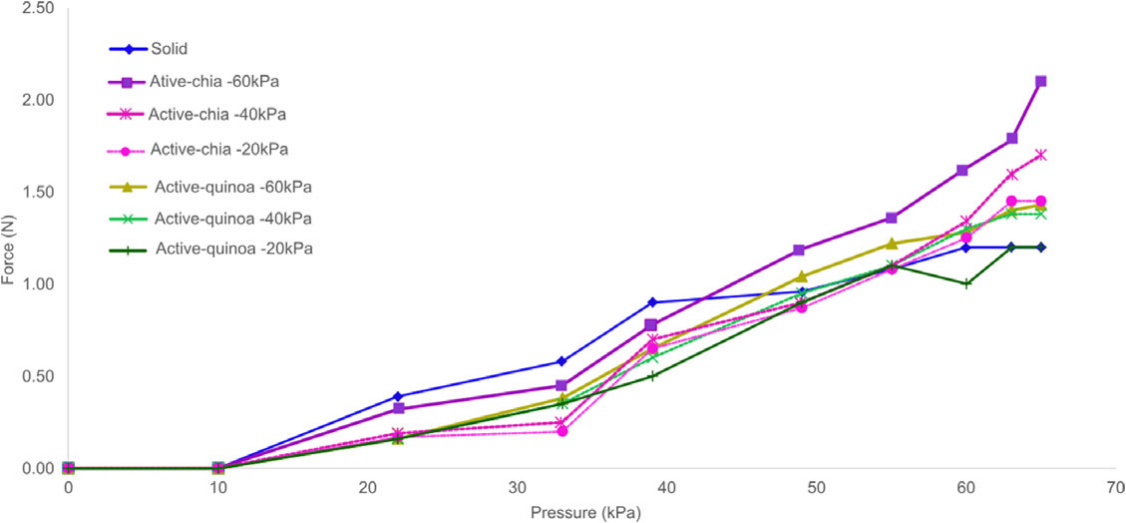
Force exerted by the actuators during the finger flexo-extension process.

Figure 16 shows the measured exerted force in the positive pressure range of 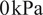 to 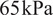 for the solid inextensible layer, active chia, and active quinoa actuators.

The actuator with the solid inextensible layer configuration achieved an average force of 1.2 N with a standard deviation of 0.38 N. The actuators with granular jamming, using chia and quinoa, exhibited different force outputs at three vacuum levels. The active chia configuration reached maximum average forces of 1.45 N, 1.7 N, and 2.1 N at 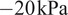 , 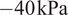 , and 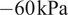 , respectively. For this configuration, there was no significant variation between the 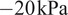 and 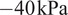 vacuum levels, with standard deviations of 0.33 and 0.35 (N), respectively. However, at 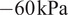 , the standard deviation increased slightly to 0.41 N. In contrast, the active quinoa actuator achieved maximum average forces of 125 N, 1.2 N, and 1.38 N at 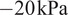 , 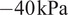 , and 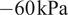 , respectively. This configuration exhibited greater variation in the measurements compared to the other two configurations. The computed standard deviations for the negative pressure values of −20 kPa, −40 kPa, and −60 kPa were 0.42, 0.45, and 0.58 (N), respectively.

The measurements show relatively high deviation standards, reflecting the inherent variability in the performance of elastomeric soft actuators with granular jamming, particularly when evaluating force and stiffness modulation. This variability arises from factors such as the nonlinear properties of the elastomeric material, inconsistencies in granular material packing, and the interaction between the actuator’s geometry and the granular medium during actuation.

**Figure 17.**
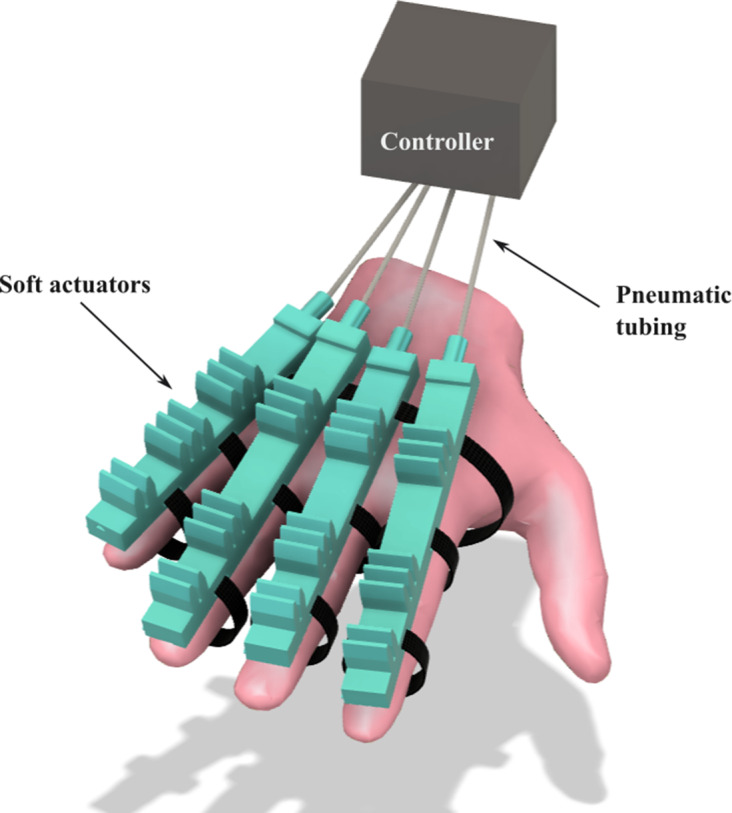
Conceptual design of the soft exoskeleton.

The soft actuator presented was parametrically designed and the index finger follows the same anthropomorphic configuration as the other large fingers. This allows for the design of a soft exoskeleton hand based on the user’s phalangeal and link dimensions. However, as future work, we propose designing the soft exoskeleton hand in standard sizes – small, medium, and large. Figure 17 illustrates the concept of soft exoskeleton hand overlaid on a virtual hand.

## 6. Conclusions

This study presents the design and characterization of a bioinspired soft pneumatic actuator utilizing granular jamming for stiffness modulation, specifically aimed at mimicking the flexo-extension motion of a human index finger for potential medical rehabilitation applications. Stiffness modulation, a core innovation of this study, plays a crucial role in enhancing the adaptability and functionality of the actuator, allowing it to perform tasks requiring variable resistance. By integrating distinct chamber geometries and natural grains for both active and passive jamming, the actuator demonstrated superior force output and stiffness modulation capabilities compared to the classic solid inextensible layer configuration, essential for applications like rehabilitation and assistive technologies.

Among the geometries evaluated, the honeycomb structure most closely replicated the finger’s natural trajectory, outperforming rectangular and half-round geometries in terms of trajectory accuracy and joint performance. The innovative combination of chamber geometry, bending motion, and stiffness modulation directly addresses challenges related to achieving natural trajectories and adaptive stiffness, making the honeycomb chamber geometry highly effective for rehabilitation tasks and human–robot interaction.

The integration of granular jamming significantly enhanced the actuator’s performance by enabling dynamic stiffness modulation, allowing for precise adjustments in resistance and force output. The active chia configuration demonstrated exceptional performance, balancing flexibility, stiffness, and trajectory alignment. This adaptability is critical for rehabilitation tasks requiring safe human interaction and reliable motion replication.

Hysteresis analysis revealed distinct inflation and deflation behaviors in the silicone-based soft actuators, forming a hysteresis loop where deflation occurred more rapidly than inflation. This characteristic enables the actuators to return to their desired positions quickly during pressure release, improving responsiveness. While the solid inextensible layer configuration followed a typical hysteresis cycle, the granular jamming configurations – particularly the passive-chia setup – showed variable behavior at lower pressure levels, introducing some performance inconsistencies. These findings emphasize the importance of stiffness modulation in maintaining reliability and adaptability during complex tasks.

Experiments also revealed that while the solid inextensible layer achieved higher force output at pressures up to 35 kPa, this was insufficient for fully bending the actuator. Granular jamming, despite adding complexity in manufacturing and actuation, provided significant advantages in stiffness modulation, enabling the actuator to adjust to different levels of resistance dynamically. The active chia configuration consistently delivered a superior performance, balancing force and stiffness while closely aligning with the index finger’s trajectory. However, proper attachment of the device to the hand is critical to prevent undesired curvatures or misalignment between the actuator and the finger.

When compared to state-of-the-art soft pneumatic actuators, such as those developed by Mészáros and Sárosi (2023) and Aktaş et al. (2021), the actuator presented in this study demonstrates superior force output and stiffness modulation. Specifically, our actuator achieves a maximum force of 2.1*N* at a positive pressure of 65 kPa for bending motion and −60 kPa for the active chia granular jamming configuration. This performance is further enhanced by its dynamic stiffness modulation capability.

In comparison, the actuator by Mészáros and Sárosi (2023) achieves a maximum force of 1.7 N at a much higher pressure of 300 kPa, utilizing passive attachable interchamber plates. This stark contrast underscores the efficiency of our design in generating higher forces at significantly lower pressures. Similarly, the actuator developed by Aktaş et al. (2021) reaches a maximum force of 1.6 N using granular jamming, further highlighting the superior performance of our approach in both force generation and adaptability.

While excelling in flexibility and adaptability, the actuator requires regulation for both positive and negative pressure to ensure optimal performance.

Despite the variability observed in some experimental results, the actuator consistently achieved controlled bending, demonstrated distinct hysteresis characteristics, and exhibited reduced variability in the active chia configuration. To improve reliability, future work will focus on optimizing material selection, enhancing granular material consistency, and refining the inextensible layer design to minimize nonuniform deformations.

Future research will build on these findings by developing a soft exoskeleton for complete hand rehabilitation, incorporating a multidirectional force measurement system for comprehensive force analysis. This will provide valuable insights into the interaction between the device and the user. Additionally, controlled human validation experiments, including subjective usability feedback and functional tests, will be conducted to evaluate the device’s assistance capabilities.

The integration of stiffness modulation as a core feature highlights the actuator’s potential for addressing complex challenges in medical rehabilitation and assistive robotics. This work represents a significant step toward creating more robust, adaptable, and user-friendly wearable technologies, advancing the fields of medical rehabilitation and human–robot interaction.

## Data Availability

Data sharing is not applicable to this article as no new data were created or analyzed in this study.
